# Twin-Screw Extrusion Desulfurized Crumb Rubber Modified Asphalt: High-Temperature Rheology and Viscoelastic Properties

**DOI:** 10.3390/ma19153227

**Published:** 2026-07-29

**Authors:** Hongying Zhang, Hongqi Zhao, Changjian Fu, Jingzhuo Zhao, Rui Dong, Jihong Han, Bo Li, Tongzhi Wang

**Affiliations:** 1 School of Civil and Hydraulic Engineering, Lanzhou University of Technology, Lanzhou 730050, China; zhystar0770@163.com; 2 Gansu Highway Traffic Construction Group Co., Ltd., Lanzhou 730030, China; 3 Gansu Province Transportation Planning Survey & Design Institute Co., Ltd., Lanzhou 730030, China; 13893329103@139.com (J.Z.); 18993250394@139.com (R.D.); 15393398907@139.com (T.W.); 4 Gansu Industry Technology Center of Transportation Construction Materials Research and Application, Lanzhou Jiaotong University, Lanzhou 730070, China; 17393183702@163.com (J.H.); libolzjtu@hotmail.com (B.L.)

**Keywords:** road engineering, desulfurized crumb rubber modified asphalt, twin-screw extrusion, high-temperature rheology, viscoelastic properties

## Abstract

To improve the efficiency of waste tire rubber powder modification in asphalt and its high-temperature performance, desulfurized rubber powder with different solubilities was prepared using a twin-screw extrusion process. Desulfurized rubber powder-modified asphalt was then produced using three types of base asphalt, Shell 90#, Zhenhai 90#, and GS 90#, as the base asphalt matrix. Dynamic shear rheometry (DSR), multi-stress creep recovery (MSCR), frequency scanning, Black curves, and complex modulus master curves were used to investigate the effects of rubber powder solubility. We focused on the complex shear modulus (G*), phase angle (*δ*), rut factor (|G*|/sin *δ*), creep recovery rate (*R*), and irreversible creep modulus (J_nr_). The results indicate that desulfurization via twin-screw extrusion effectively breaks the sulfur cross-links in the rubber powder, as inferred from the significant increase in solubility, significantly improving rubber powder-asphalt compatibility. Of the three modified asphalts, Shell 90# desulfurized rubber powder-modified asphalt exhibited the slowest decay in high-temperature complex shear modulus, the smallest increase in phase angle, and the best high-temperature rutting factor stability. MSCR tests further confirmed that Shell 90# desulfurized rubber powder-modified asphalt exhibited the highest creep recovery rate, the lowest irreversible creep modulus, and the greatest resistance to permanent deformation. The black curve and the complex modulus master curve confirm that Shell 90# modified asphalt has the best viscoelastic balance and the most stable microstructure. These findings provide a theoretical basis for designing rubber-modified asphalt pavement materials for use in regions with high temperatures and heavy rainfall.

## 1. Introduction

With the rapid development of the transportation industry, the total length of expressways and urban roads continues to grow. Due to the combined effects of traffic loads, high-temperature radiation, and rain erosion, asphalt pavements are prone to permanent deformation defects such as rutting, heaving, and potholes, which severely compromise road service life and traffic safety [1,2]. Asphalt modified with recycled tire rubber powder can significantly improve the asphalt’s high-temperature stability, low-temperature crack resistance, and fatigue performance, while simultaneously facilitating the resource recovery of solid waste, aligning with the development direction of green and low-carbon road engineering [3,4].

However, traditional rubber powder has an inert surface, poor compatibility with asphalt, and uneven dispersion within the asphalt matrix. Additionally, its cross-linked structure is excessively dense, leading to significant fluctuations in the high-temperature rheological properties of the modified asphalt and insufficient storage stability [5,6,7]. Existing research indicates that the rubber molecular chains in waste vulcanized rubber powder form a three-dimensional network that is difficult to effectively dissociate in asphalt [8,9]. Desulfurization and activation technology, by breaking the S-S and C-S bonds within the three-dimensional network of vulcanized rubber, allows for the controlled adjustment of cross-linking density [10,11,12]. This enhances the surface activity and swelling capacity of the rubber powder, generates active groups on its surface, and improves its compatibility with asphalt, making it a key solution to the aforementioned issues. Among these methods, twin-screw extrusion stands out as a highly promising pre-treatment technique for internal activation of waste rubber powder. By utilizing mechanical forces and friction, it alters the structure and morphology of the rubber powder, enabling the controlled breaking and activation of its cross-linked network [13].

In recent years, scholars both domestically and internationally have conducted extensive research on the preparation process, microstructure, and road performance of desulfurized rubber powder-modified asphalt [5,6,14]. Chen et al. investigated the swelling mechanism and road performance of desulfurized rubber asphalt, finding that desulfurization treatment significantly improves the dispersion state and swelling behavior of rubber powder in asphalt [15]. Yan et al. employed twin-screw extrusion to desulfurize rubber powder at different temperatures, finding that the extrusion temperature significantly affects the sol content of the rubber powder as well as rheological parameters of the modified asphalt, such as the complex shear modulus and phase angle [16]. Wu et al. compared three activation processes, microwave, chemical, and twin-screw extrusion, using various methods such as Fourier transform infrared spectroscopy, scanning electron microscopy, and thermogravimetric analysis and found that twin-screw extrusion activation can significantly break polysulfide cross-links through strong shear action, thereby improving the solubility of rubber powder [17]. Regarding rheological characterization methods, dynamic shear rheometry and multi-stress creep-recovery tests have been widely used to study the high-temperature rheological properties and high-temperature deformation resistance of desulfurized rubber powder-modified asphalt, while frequency scanning and Black curve analysis are important tools for evaluating the viscoelastic properties and structural stability of modified asphalt [18].

However, existing studies have primarily focused on the effects of activation methods or process parameters on the performance of modified asphalt in single-base asphalt systems. Systematic comparative studies on the high-temperature rheological behavior of desulfurized rubber powder with different solubilities in various grades of base asphalt remain scarce. In particular, a comprehensive analytical framework that combines DSR temperature scanning, MSCR, Black curve analysis, and complex modulus master curves is still lacking. The temperature range of 52–76 °C selected in this study is based on the typical high-temperature conditions encountered in asphalt pavements in regions with high temperatures and heavy rainfall, such as Northwest China, where surface temperatures can reach 60–70 °C during summer months. The high-temperature rheological parameters employed in this study-including complex shear modulus (*G*), phase angle (*δ*), rutting factor (|*G*|/sin *δ*), creep recovery rate (*R*), and irreversible creep compliance (J_nr_)-are well-established performance indicators that have been correlated with field rutting behavior through the Superpave performance grading system and AASHTO M332 specifications. Therefore, the laboratory measurements in this study provide a reliable basis for predicting the relative high-temperature performance of different modified asphalt formulations in actual pavement applications. Accordingly, this study employs a twin-screw extrusion process to prepare desulfurized rubber powders with varying solubilities. Three typical 90# road asphalts, Shell 90#, Zhenhai 90#, and GS 90#, were selected as base asphalts. The high-temperature rheological parameters of the desulfurized rubber powder-modified asphalts were systematically tested. By analyzing the decay patterns of the complex shear modulus, the evolution trends of the phase angle, rutting factor stability, creep recovery capacity, and irreversible creep modulus, this study reveals the intrinsic relationship between rubber powder solubility and base asphalt type on high-temperature rheological behavior. This study aims to establish a comprehensive rheological performance evaluation system by integrating Black curves with complex modulus master curves, thereby providing scientific support for the engineering application of high-performance desulfurized rubber asphalt.

## 2. Materials and Methods

### 2.1. Raw Materials

#### 2.1.1. Recycled Rubber Powder

40-mesh CR produced by ambient grinding was used. The basic technical indexes are given in Table 1.

#### 2.1.2. Activation Additives and Compatibilizer

The molecular penetration of extracted oil induces volume expansion of the rubber powder and enhances its surface activity, thereby optimizing the kinetics of the desulfurization reaction while reducing the interfacial tension between the polymer and asphalt. Therefore, extracted oil is used as an activator for rubber powder and a compatibilizer for modified asphalt. It is a brownish-yellow viscous liquid with a relative density of 1.02 g/cm^3^ (20 °C), a viscosity of 50 cp, a flash point of 220 °C, and an aromatic content of 88%.

Dibenzyldisulfide (DBDS) was selected as the activation aid; it is a white crystalline powder with a melting point of 69~72 °C, a density of 1.30 g/cm^3^, and a flash point of 235 °C. Under thermal-mechanical conditions, DBDS undergoes homolytic cleavage to generate active sulfur radicals, which controllably break the S-S bonds in the three-dimensional network of the rubber powder, thereby enabling precise regulation of the vulcanization crosslinking density.

#### 2.1.3. Base Asphalts

Three types of road petroleum asphalt, Shell 90# (QP 90#), Zhenhai 90# (ZH 90#), and GS 90#, were selected as base asphalt. Technical specifications were tested in accordance with the “Test Procedures for Asphalt and Asphalt Mixtures in Highway Engineering” (JTG E20-2011) [20]. Taking Shell 90# as an example, the test results for its technical specifications are shown in Table 2. The technical specifications of the other two asphalts also meet the requirements of the 90# road asphalt standard.

### 2.2. Test Methods

#### 2.2.1. Preparation of Desulfurized Rubber Powders with Different Solubilities

The twin-screw extrusion method activates rubber powder through mechanical compression and friction. It is a relatively mild activation method that primarily relies on mechanical forces to alter the structure and morphology of the rubber powder, without involving high-temperature chemical reactions. In this study, an XH-433-40 twin-screw extruder (Guangdong Xihua Machinery Co., Ltd., Dongguan, China, screw diameter 25 mm, length-to-diameter ratio 40:1) was used for rubber powder activation.

Key process parameters: five-zone temperature control with a gradient ranging from 80 to 160 °C, screw speed of 200–400 r/min, and feed rate of 10 kg/h. The activation process consists of two stages: ① Pretreatment: 500 g of raw rubber powder was dried at 50 °C for 1 h, then mixed with DBDS and extracted oil (heated to 80 °C) in the specified ratio. The mixture was stirred at high speed for 5 min and allowed to stand for 24 h to ensure thorough penetration of the extracted oil. ② Twin-screw extrusion: After 24 h of pretreatment, the rubber powder is loaded into a twin-screw extruder preheated to the extrusion temperature, where the feed screw conveys the powder to the main screw for extrusion. The activated rubber powder is collected after the equipment has operated stably for 5 min. To prevent fire caused by heat dissipation from powder accumulation, timely stirring is required during the experiment. After extrusion, the equipment is shut down, and the product is cooled in a fume hood before storage.

The solubility of the rubber powder is determined using the Soxhlet extraction method, with the degree of desulfurization characterized by the percentage of sol components that have lost their vulcanized network relative to the total mass of the rubber powder. The solubility of the activated rubber powder is calculated according to Equation (1):

(1) $$Sol\%=(1-\frac{m_{3}-m_{1}}{m_{2}-m_{1}})\times 100\%$$

**Figure 1:**
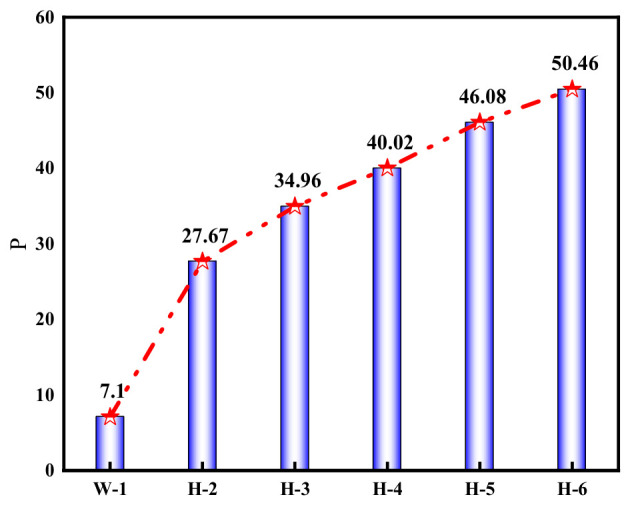
Solubility of Activated Rubber Powder in a Twin-Screw Extruder.

Two samples of the same desulfurized powder were taken, and the average solubility was calculated as the final solubility of the powder. See Figure 1 for details.

The relationship between solubility and crosslink density can be theoretically described by the Flory-Rehner theory [21], which relates the polymer volume fraction in the swollen state to the crosslink density of the vulcanized network. According to this theory, the sol fraction (solubility) is inversely related to the crosslink density: higher solubility values indicate a greater proportion of polymer chains that have been released from the three-dimensional vulcanization network, corresponding to lower crosslink density. In this study, the solubility values of the activated rubber powders were measured as: W-1 (unactivated): 7.1%, H-2: 27.67%, H-3: 34.96%, H-4: 40.02%, H-5: 46.08%, and H-6: 50.46%. The monotonic increase in solubility with increasing extrusion severity is consistent with a progressive decrease in crosslink density. While these solubility values do not directly provide crosslink density in units such as mol/cm^3^, they provide a reliable and reproducible measure of the relative extent of desulfurization. In future studies, direct crosslink density measurements, such as equilibrium swelling tests or nuclear magnetic resonance (NMR) spectroscopy, will be performed to establish a more precise quantitative relationship between solubility and crosslink density in desulfurized rubber powder systems.

It is important to acknowledge that solubility, as measured by the Soxhlet extraction method, serves as a macroscopic indicator of the degree of desulfurization but does not fully characterize the extent of devulcanization at the molecular level. The cleavage of S-S and C-S bonds, which is the fundamental mechanism of desulfurization, requires direct chemical characterization for confirmation. While the increase in solubility is consistent with the disruption of sulfur crosslinks, it should be noted that solubility alone does not provide direct molecular-level evidence of S-S bond cleavage. In future studies, complementary techniques including Fourier-transform infrared spectroscopy (FT-IR), X-ray photoelectron spectroscopy (XPS), and Raman spectroscopy will be employed to provide direct experimental evidence of sulfur bond cleavage and to elucidate the desulfurization mechanism.

#### 2.2.2. Preparation of Desulfurized Rubber Powder-Modified Asphalt

Desulfurized rubber powder-modified asphalt was prepared using a shear-mixing unit (integrated with a mixer, high-speed shear disperser, and electric heating jacket). The specific steps are as follows: First, the base asphalt was heated to 135 °C until completely melted; 500 g was then poured into an asphalt tank, placed in a heat-retaining jacket, and continuously heated to the target temperature while being stirred until uniform. Next, weigh the activated rubber powder and compatibilizer according to the following formulation: rubber powder content of 20% by weight of base asphalt, compatibilizer (extracted oil) content of 10% by weight of rubber powder, DBDS content of 1.5% by weight of rubber powder, and stabilizer content of 0.4% by weight of total mixture. Add them to the asphalt while the mixer is running at 1000 r/min. Set the electric heating jacket temperature to 185 °C, stir for 30 min, then increase the speed to 5000 r/min for high-speed shearing for 1 h. After shearing, pour the mixture back into the mixer, add 0.4% stabilizer, and continue stirring at 1000 r/min for 2.5 h to obtain the final modified asphalt.

#### 2.2.3. Test Methods for Rheological Properties

(1) Dynamic Shear Rheometer (DSR) Testing

A Discovery HR-20 DSR testing system was used to characterize the high-temperature rheological properties of rubber-modified asphalt, primarily involving three-stage rheological testing:

Temperature sweep

In accordance with ASTM D7405-2020 [22], a 25 mm parallel-plate fixture was used with a temperature gradient of 52–76 °C in 6 °C increments, an angular frequency of 10 rad/s, a strain of 12%, and a gap of 1 mm. The viscoelastic parameters were calculated using Equations (2)–(4).

(2) $$G^{*}=G^{\prime }+iG^{\prime \prime }$$

(3) $$G^{\prime }=\left|G^{*}\right|*\cos\delta$$

(4) $$G^{\prime \prime }=\left|G^{*}\right|*\sin\delta$$

where $G^{\prime }$ is the storage modulus; $G^{\prime \prime }$ is the loss modulus.

(2) Multiple Stress Creep and Recovery (MSCR) Test

In accordance with ASTM D8239-23 [23], this test quantitatively characterizes the nonlinear viscoelastic properties of materials through their creep-recovery behavior at dual stress levels of 0.1 kPa and 3.2 kPa. In each cycle, each stress level consists of 10 cycles (1 s loading/9 s unloading).

(3) Frequency sweep

Given the high incidence of rutting in asphalt pavements within the 40–60 °C temperature range, a dynamic frequency sweep from 0.1 to 100 Hz was conducted at 60 °C with a strain of 1% [24].

All rheological tests, including temperature sweep, MSCR, and frequency sweep, were performed on three independently prepared specimens for each modified asphalt formulation. The reported results represent the mean values of these three replicates to ensure data reliability and reproducibility.

## 3. Analysis of High-Temperature Rheological Properties Based on DSR

This study investigated three types of base bitumen, QP 90#, ZH 90#, and GS 90#, and used a dynamic shear rheometer system to test the high-temperature rheological behavior of desulfurized rubber powder-modified bitumen with varying solubility. Through temperature scanning, the temperature-dependent variations in the complex shear modulus, phase angle, and rutting factor were obtained. Based on MSCR tests, the creep recovery rate and irreversible creep modulus were calculated to evaluate the material’s resistance to permanent deformation in both linear and nonlinear viscoelastic regions. Furthermore, frequency scans, Black curves, and complex modulus master curves were utilized to analyze the viscoelastic frequency dependence and structural stability characteristics of the modified asphalt. By integrating these rheological parameters, the study systematically elucidated the mechanisms by which rubber powder solubility and base asphalt type influence high-temperature rheological behavior, thereby providing a basis for selecting desulfurized rubber powder-modified asphalt systems with superior high-temperature performance.

### 3.1. Temperature Sweep

**Figure 2:**
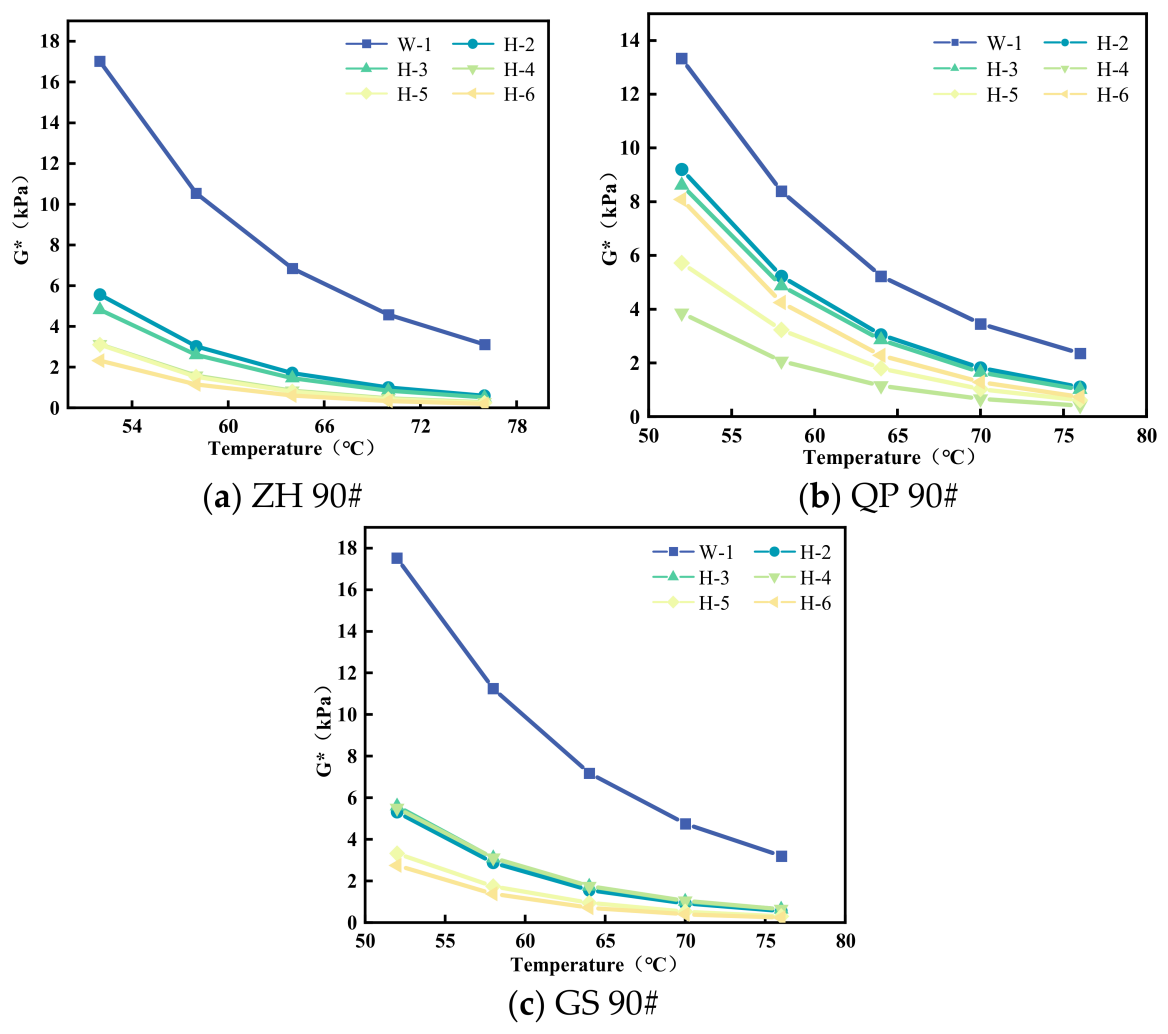
Complex shear modulus G*.

Due to the viscoelastic nature of asphalt, a response lag occurs under sinusoidal loading. This degree of lag is defined as the phase angle *δ*, whose value ranges between 0° for an ideal solid and 90° for an ideal fluid. Figure 2 shows the variation in G* for various modified asphalts as a function of relevant factors at different dissolution levels.

(1) Complex shear modulus G*

During the frequency scan ranging from 0.1 to 100 rad/s, the G* values of the different activated rubber powder-modified asphalts varied significantly. As shown in Figure 2, the G* values of all three asphalts decreased with increasing temperature, indicating that heating enhances the material’s fluidity. Specifically, Figure 2a shows that the G* value of ZH 90# modified asphalt decreased significantly with rising temperature. W-1 decreased from 17.01 kPa to 3.12 kPa, a reduction of approximately 82%, which is attributed to insufficient high-temperature stability due to low polymer crosslinking density [25]; whereas the solubility increased from H-3 to H-6, the dispersion of the rubber powder improved, a crosslinked network structure gradually formed, and deformation resistance increased, resulting in a flatter trend in the decrease in G* at high temperatures and a more pronounced inhibitory effect on flow.

Figure 2b shows that although the G* value of QP 90# modified asphalt decreased overall from 13.318 kPa to 2.36 kPa, a reduction of approximately 82.3%, the decline was relatively gradual. Its moderate degree of molecular chain cross-linking and high thermal stability enable the material to maintain strong resistance to deformation at high temperatures, with minimal fluctuations in the G* value, demonstrating excellent high-temperature stability. The cross-linking structure of the rubber powder provides a more balanced regulation of the asphalt’s flow properties. Figure 2c shows that the G* of GS 90# modified asphalt fluctuates dramatically with increasing temperature, and its high-temperature performance deteriorates rapidly; for example, at W-1, it drops from 17.51 kPa to 3.198 kPa, a decrease of approximately 81.8%. This is attributed to excessive cross-linking of the polymer chains in the rubber powder, which leads to poor high-temperature flowability; insufficient cross-linking at low solubility results in poor stability; while high solubility can increase the degree of cross-linking, excessive cross-linking conversely causes greater fluctuations in G* at high temperatures, leading to an imbalance in flowability regulation [26].

A comprehensive comparison shows that QP 90# desulfurized rubber powder-modified asphalt exhibits the most stable performance at high temperatures, with a gradual change in G* value and the ability to effectively maintain high deformation resistance. This is closely related to its appropriate degree of molecular chain cross-linking and good thermal stability. GS 90# modified bitumen exhibits relatively drastic changes in G* values in both low- and high-temperature ranges, with a rapid decline in high-temperature performance. This is attributed to poor high-temperature flow properties caused by excessive cross-linking of the polymer molecular chains. ZH 90# modified asphalt exhibits poor high-temperature performance and high-temperature sensitivity upon cooling, making it prone to deformation in high-temperature environments; the adverse effects of the cross-linked structure under high-temperature conditions reduce the asphalt’s flowability, further weakening its high-temperature stability. Overall, QP 90# demonstrates the best high-temperature rheological properties among the three modified asphalts due to the optimized balance between its cross-linked structure and viscoelasticity.

(2) Phase angle (*δ*)

**Figure 3:**
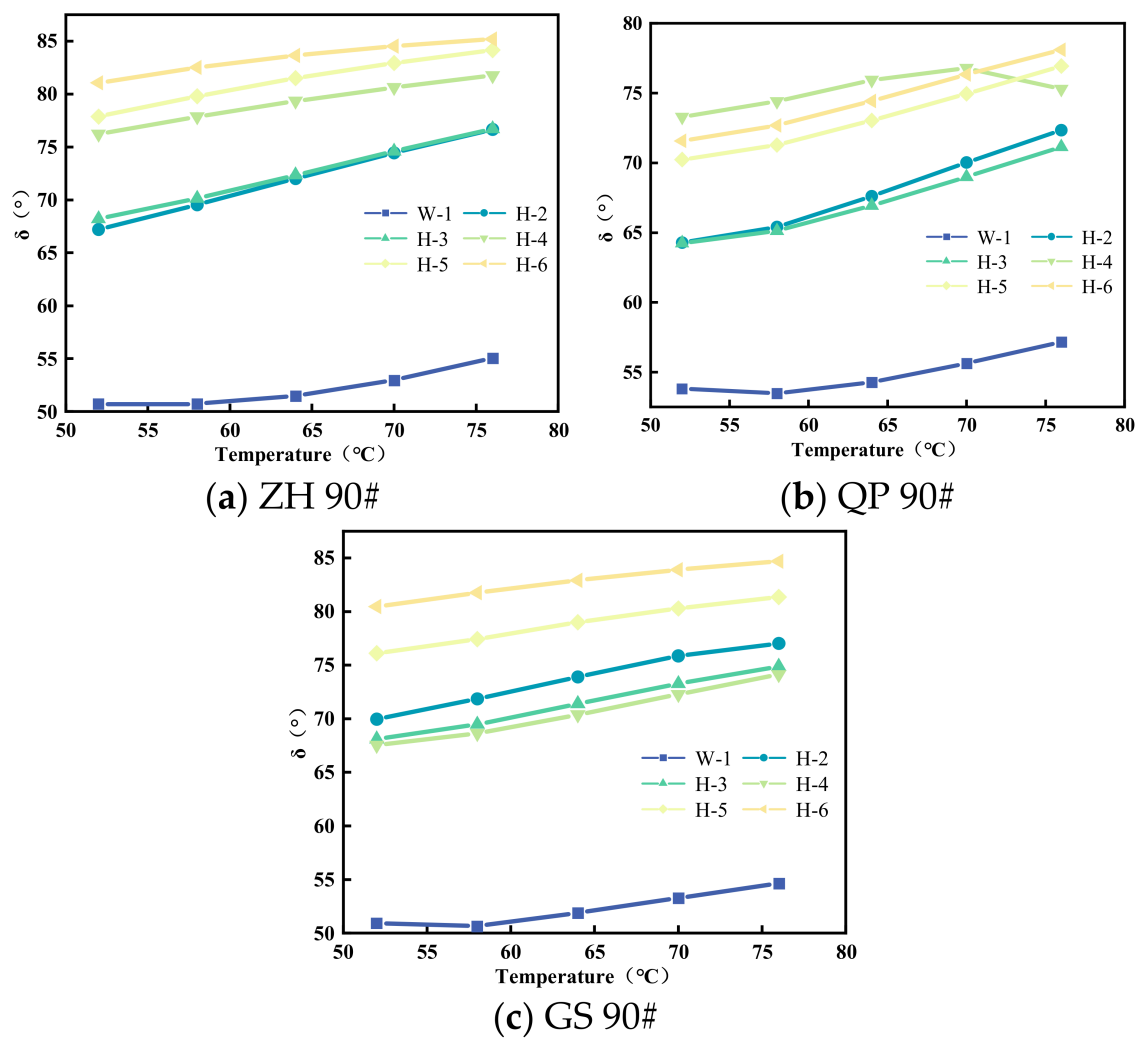
δ values of modified asphalt.

*δ* is an important parameter that describes the balance between a material’s elastic and viscous responses. A smaller phase angle typically indicates that the material exhibits stronger elastic properties (solid-like behavior), while a larger phase angle suggests that the material exhibits more viscous characteristics (liquid-like behavior). Figure 3 shows the variation in δ for various modified asphalts at different solubility levels.

*δ* is used to characterize the balance between a material’s elastic and viscous responses. The smaller the δ value, the more the material tends toward elasticity (similar to a solid); conversely, the larger the *δ* value, the more pronounced the viscous characteristics (similar to a fluid). Figure 3 shows the trend in *δ* values for various modified asphalts at different dissolution levels.

As shown in Figure 3a, the phase angle of ZH 90# modified asphalt (W-1, H-2 to H-6) reached 53.47% during the temperature rise from 50 °C to 80 °C, indicating that the material’s viscosity increased and its elasticity decreased as the temperature rose. However, the increase in phase angle was not consistent at the same solubility; H-6 had a phase angle approaching 85° in the high-temperature range of 75–80 °C, indicating that its viscous response predominated, while its elasticity was relatively weak. Figure 3b shows that the phase angle of H-6 in QP 90# modified bitumen exceeds 75° in the same high-temperature range, exhibiting distinct viscous characteristics; the phase angle of the remaining samples increases more gradually, maintaining a good balance between elasticity and viscosity, and demonstrating good high-temperature stability. As shown in Figure 3c, the phase angle of GS 90# modified bitumen increased by as much as 66.15% in the 50–80 °C range. This is due to high solubility, which results in more uniform dispersion of the rubber powder and a strengthened cross-linked network, leading to a significant increase in the viscous component but a pronounced loss of elasticity [27]. Although this change is beneficial for resisting high-temperature deformation, it may lead to excessive fluidity and insufficient elasticity. Considering the overall trends in phase angle evolution and performance at different temperatures, QP 90# modified asphalt demonstrates superior viscoelastic properties.

A comparative analysis of the three asphalts reveals that GS 90# modified asphalt exhibits a phase angle increase of 66.15%. Due to its high solubility, the rubber powder disperses well, and the cross-linked structure is dense, resulting in a marked increase in viscosity and a corresponding decrease in elasticity. The increase for ZH 90# was 53.47%; while viscosity increased, elasticity was significantly reduced, and fluidity was relatively high. QP 90# showed the smallest increase at only 45.39%, demonstrating the optimal balance between elasticity and viscosity. It is particularly stable under high-temperature conditions and exhibits the most reasonable overall viscoelastic properties.

(3) Rutting factor (|G*|/sin δ)

**Figure 4:**
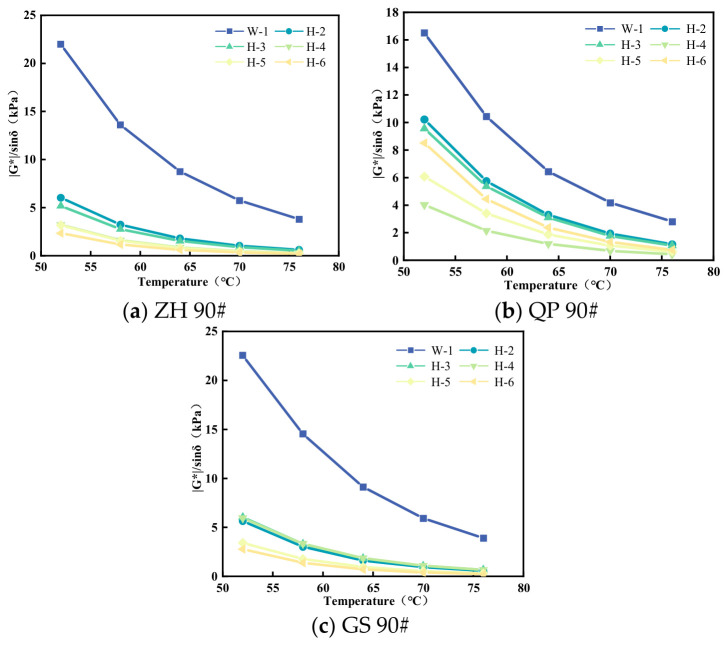
Schematic diagram of |G*|/sin *δ*.

In the SHRP program, |G*|/sin δ is used to characterize the resistance of asphalt to high-temperature permanent deformation, i.e., the rutting factor. Figure 4 shows the trend of |G*|/sin δ for modified asphalt at different solubility levels.

As shown in Figure 4a, the rutting factors of asphalt modified with rubber powder of different solubilities exhibit varying patterns of change with temperature. For ZH 90# modified asphalt, the rutting factor decreases continuously as the temperature rises, with a reduction of approximately 82% in the range from 52 °C to 76 °C, indicating a significant weakening of rutting resistance at high temperatures. This is attributed to the high content of long-chain polymers in the rubber powder, which provides significant modification effects at low temperatures [28]. However, thermal expansion at high temperatures causes the rubber powder structure to gradually loosen, leading to a rapid decline in the rutting factor. As shown in Figure 4b, the rutting factor of QP 90# is lower than that of ZH 90# at all temperatures. This is attributed to the better dispersion of low-molecular-weight polymers in the QP 90# rubber powder, which results in good low-temperature performance and stronger structural retention at high temperatures, leading to a slower decline in the rutting factor [29]. Figure 4c shows that the trend of the rutting factor of GS 90# rubber powder-modified asphalt with temperature is similar to that of QP 90#, but its low-temperature performance is superior, with a rutting factor of 22.56 at 52 °C and 3.92 at 76 °C. Although the high-temperature value is lower than the low-temperature value, it still outperforms the performance of ZH 90# at the same temperatures, demonstrating strong overall rutting resistance and good adaptability across different temperature ranges.

In summary, the rutting factors of asphalt modified with rubber powder of different solubilities exhibit significant temperature dependence. ZH 90# experiences a sharp decline in rutting factor at high temperatures, resulting in a rapid decrease in rutting resistance. QP 90# shows a gradual change in rutting factor, with outstanding high-temperature rutting resistance and balanced overall performance. GS 90# exhibits a high rutting factor at low temperatures; although it decreases somewhat at high temperatures, it still maintains a certain advantage in rutting resistance. Among these, QP 90# leverages the advantages of its polymer structure to effectively maintain rutting resistance under high-temperature conditions while meeting multi-temperature requirements, making it the optimal and most balanced choice for resistance to high-temperature permanent deformation. Meanwhile, GS 90# demonstrates excellent low-temperature performance and relatively stable high-temperature performance. ZH 90# suffers from significantly reduced rutting factors at high temperatures, limiting its rutting resistance. Overall, QP 90# is the most advantageous choice, offering both balanced performance and superior high-temperature properties.

### 3.2. MSCR Analysis

**Figure 5:**
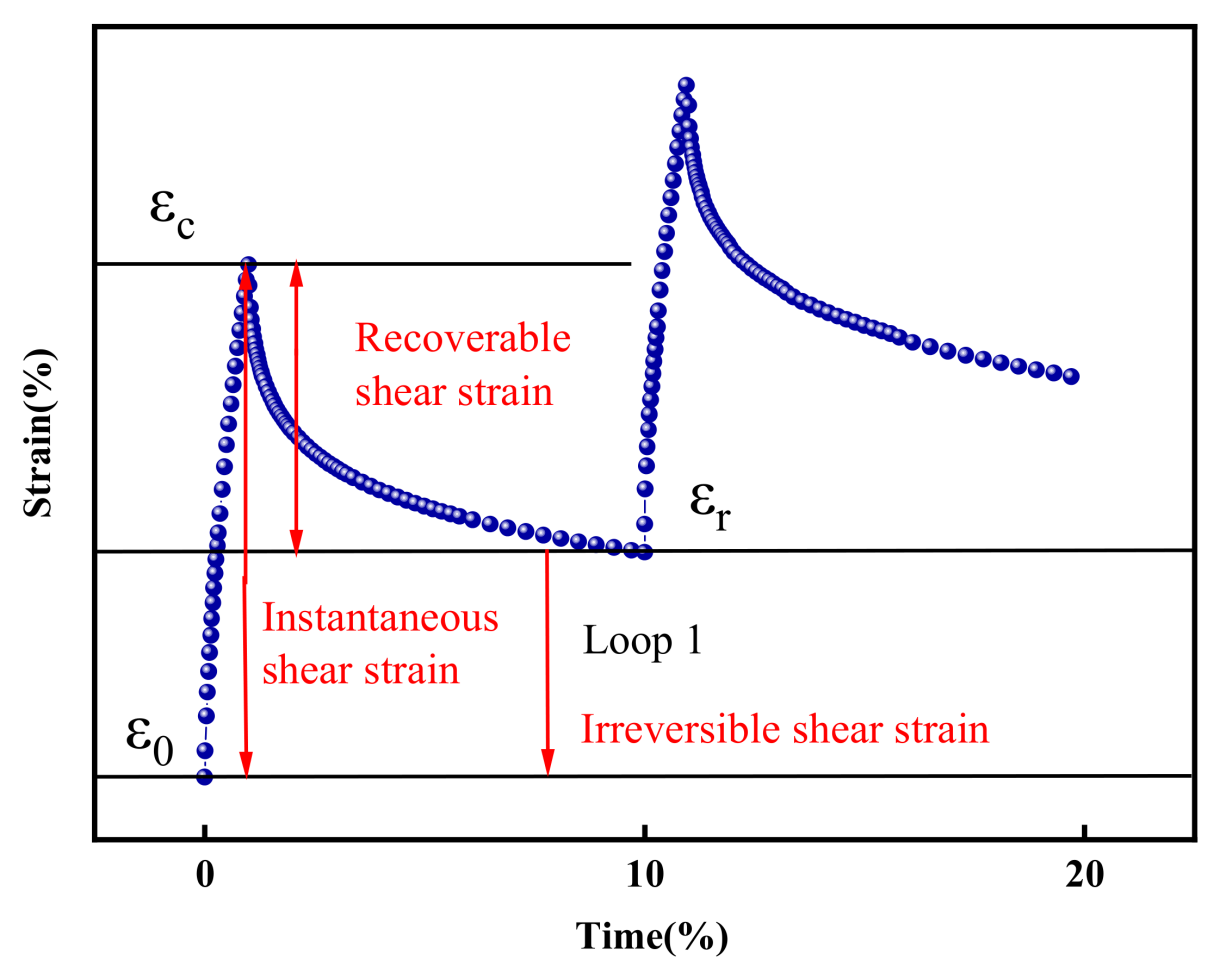
Strain-stress relationship during the MSCR process.

In the Superpave framework, G*/sin*δ* is commonly used to evaluate the high-temperature performance of asphalt; however, this parameter only reflects the material’s behavior within the linear viscoelastic range. In contrast, the MSCR test separates permanent deformation from total deformation, providing a more accurate representation of the evolution of asphalt’s viscoelastic properties during loading. Additionally, its creep-recovery loading regimen effectively simulates the formation of rutting in pavements. The test employs two stress levels: 0.1 kPa to characterize linear viscoelastic response, and 3.2 kPa to evaluate nonlinear viscoelastic behavior. The primary evaluation indices include the average recovery rate (R) and the average irreversible creep modulus (J_nr_). The former reflects the asphalt’s elastic recovery capacity and stress dependence during loading, while the latter characterizes the degree of permanent deformation and is closely related to the pavement’s resistance to rutting [30,31]. The calculation methods for R and J_nr_ are shown in Equations (5) and (6), and the definitions of relevant parameters are illustrated in Figure 5. In this study, the MSCR test temperature range was 52 °C to 64 °C, with stress levels of 0.1 kPa and 3.2 kPa, respectively. The specific test procedure followed AASHTO M332-18 [32].

The average creep recovery rate *R* and the average irreversible creep compliance J_nr_ are calculated using Equations (5) and (6).

(5) $$R=\frac{\varepsilon_{p}-\varepsilon_{u}}{\varepsilon_{p}}\times 100\%$$

(6) $$J_{\mathrm{nr}}=\frac{\varepsilon_{u}}{\sigma }$$

In the equations, $\varepsilon_{p}=\varepsilon_{c}-\varepsilon_{0}$; $\varepsilon_{u}=\varepsilon_{r}-\varepsilon_{0}$; *ε*_0_: initial strain; *ε*_c_: strain at the end of creep; *ε*_r_: strain at the end of recovery.

At 0.1 kPa and 3.2 kPa, the creep recovery rate for a single creep cycle is calculated using Equations (7) and (8), the average creep recovery rate over 10 creep cycles was calculated using Equations (9) and (10), the irreversible creep compliance was calculated using Equations (11) and (12), and the mean irreversible creep compliance over 10 creep cycles was calculated using Equations (13) and (14).

(7) $$R_{r}(0.1,N)=\frac{\varepsilon_{p}-\varepsilon_{u}}{\varepsilon_{p}}\times 100\%,\;N=(1,10)$$

(8) $$R_{r}(3.2,N)=\frac{\varepsilon_{p}-\varepsilon_{u}}{\varepsilon_{p}}\times 100\%,\;N=(1,10)$$

(9) $$R_{0.1}=\frac{\sum_{N}^{10}\varepsilon_{r}(0.1,N)}{10},\;N=(1,10)$$

(10) $$R_{3.2}=\frac{\sum_{N}^{10}\varepsilon_{r}(3.2,N)}{10},\;N=(1,10)$$

(11) $$J_{nr}(0.1,10)=\frac{\varepsilon_{u}}{10},\;N=(1,10)$$

(12) $$J_{nr}(3.2,10)=\frac{\varepsilon_{u}}{3200},\;N=(1,10)$$

(13) $$J_{nr0.1}=\frac{\sum_{N=1}^{10}J_{nr}(0.1,N)}{10},\;N=(1,10)$$

(14) $$J_{nr3.2}=\frac{\sum_{N=1}^{10}J_{nr}(3.2,N)}{10},\;N=(1,10)$$

(1) Average Elastic Recovery Rate (*R*)

**Figure 6:**
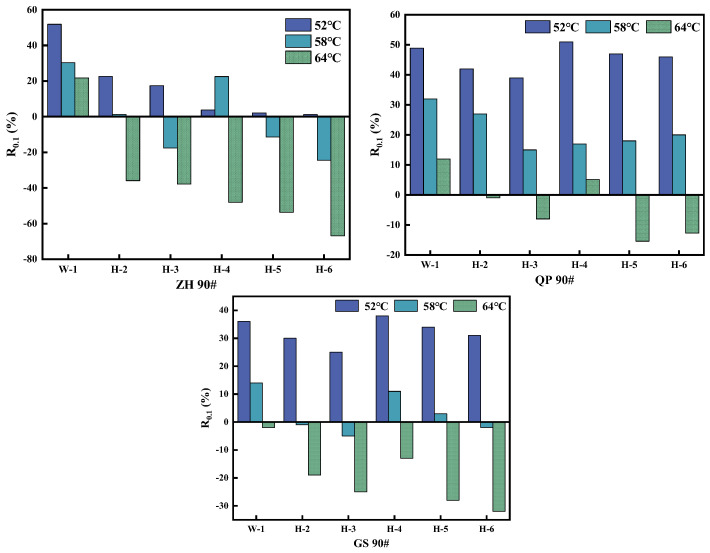
R_0.1_ Value of Rubber Powder-Modified Asphalt.

The creep recovery rate measures the ability of asphalt to recover from its original deformation after the load is removed; a higher R value indicates better resistance to rutting. Figure 6 shows the variation in the average elastic recovery rate of activated rubber powder-modified asphalt within the temperature range of 52 °C to 64 °C.

As shown in Figure 6 for the ZH 90# modified asphalt, at a stress level of 0.1 kPa, the R_0.1_ value of the unactivated sample W-1 was approximately 50% at 52 °C; after activation, this value decreased nonlinearly as solubility increased. When the temperature rises to 58 °C, R_0.1_ for W-1 drops to 30%, while that of the activated sample approaches zero or even becomes negative, reaching its highest negative value at 64 °C. This indicates that high temperatures cause viscous flow to dominate, with elastic recovery capacity nearly lost, thereby confirming the dissociative effect of activation on the rubber powder cross-linking network and the significant influence of temperature on the viscoelastic behavior of asphalt.

Negative recovery values (R < 0) observed in the MSCR test indicate that the sample undergoes further deformation rather than recovering after load removal. This phenomenon is not a measurement artifact but reflects the material’s rheological behavior when viscous flow dominates to such an extent that time-dependent creep continues even after unloading. In practical terms, negative R values indicate extremely poor elastic recovery and high susceptibility to permanent deformation, effectively meaning the material behaves as a viscous fluid rather than a viscoelastic solid. According to AASHTO M332-18, negative R values at the standard test temperature indicate that the material fails to meet the minimum elastic recovery requirements for all traffic grades, thus significantly limiting its practical application in pavement construction. The occurrence of negative recovery values in this study is attributed to the combination of high temperature (≥64 °C) and elevated rubber powder solubility, which promotes viscous flow while simultaneously diminishing the elastic contribution of the polymer network.

Analysis of the QP 90# modified asphalt in Figure 6 reveals that at 52 °C, both W-1 and R_0.1_ of the activated sample reached 55%, but its decay rate was lower than that of ZH 90#. At 58 °C, W-1 dropped to 30%, while the activated sample remained within the 10–20% range. At 64 °C, R_0.1_ remained positive for some samples, indicating a slower decline in elastic recovery capacity at high temperatures, with both rutting resistance and stability superior to those of ZH 90#. In contrast, for the GS 90# modified asphalt shown in Figure 6, both W-1 and the R_0.1_ of the activated sample were generally lower than those of QP 90# at 52 °C; at 58 °C, W-1 dropped to 15%, with activated samples concentrated between 0% and 10%, and the proportion of negative values increased at 64 °C. This indicates pronounced viscous flow characteristics at high temperatures, with elastic recovery significantly affected by temperature, and rutting resistance inferior to that of QP 90#.

In summary, all three asphalts conform to the general rule that “R_0.1_ decreases as temperature increases,” and the R_0.1_ values of activated rubber powder are generally lower than those of unactivated rubber powder, verifying the mechanism whereby activation promotes the breakdown of the rubber powder cross-linking network and enhances elastic dissipation effects. Under low-stress conditions, increased solubility causes the R_0.1_ value to exhibit nonlinear decay, which is closely related to the uneven particle size distribution of the rubber powder and the disruption of the three-dimensional network structure. Among them, QP 90# has the highest overall R_0.1_ value and the smallest high-temperature decay; it still retains some positive values at 64 °C, demonstrating the best rutting resistance. ZH 90# exhibited the highest proportion of negative values at high temperatures, the most severe loss of elastic recovery, and the poorest high-temperature performance. GS 90# fell between the two, exhibiting significant viscous flow at high temperatures, with rutting resistance inferior to that of QP 90#.

**Figure 7:**
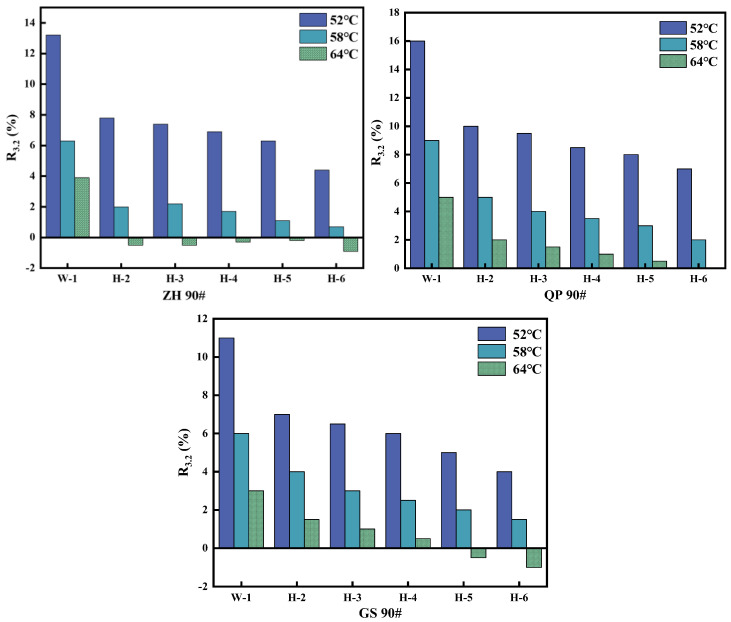
R_3.2_ Value of Rubber Powder-Modified Asphalt.

As shown in Figure 7, the R_3.2_ value of ZH 90# rubber powder-modified asphalt continues to decrease as the temperature rises; in particular, it is significantly lower at 64 °C than at 52 °C, indicating that the material’s viscous characteristics increase at high temperatures, while its elastic recovery capacity weakens. In contrast, the R_3.2_ value of QP 90# modified asphalt is generally higher, and its rate of decline with increasing temperature is lower than that of ZH 90#, suggesting that QP 90# retains a certain degree of elastic recovery capability under high-temperature conditions. The R_3.2_ value of GS 90# is slightly lower than that of ZH 90# but higher than that of QP 90#. Furthermore, its R_3.2_ value decreases gradually with rising temperature, turning negative at 64 °C, indicating that the material’s recovery capacity is extremely limited at very high temperatures, with viscous behavior becoming the dominant factor.

In summary, all three asphalts follow the common pattern of “decreasing R value with rising temperature.” This is due to increased molecular chain motion at higher temperatures, which enhances viscosity and degrades creep recovery capacity. Among them, ZH 90# exhibits the most rapid decline in recovery capacity at high temperatures; at 64 °C, viscous behavior dominates, and elastic recovery is severely weakened. QP 90# consistently exhibits the highest R_3.2_ value, with the smallest decrease at high temperatures; it remains positive at 64 °C, demonstrating the best elastic recovery performance among the three. GS 90# experiences a sharp decline in R_3.2_ at high temperatures, turning negative at 64 °C; at extremely high temperatures, viscous behavior completely dominates, and recovery capacity is virtually lost. Overall, QP 90# exhibits more balanced elastic recovery capabilities under various temperature conditions and demonstrates outstanding resistance to deformation. ZH 90# and GS 90# exhibit insufficient high-temperature performance, with GS 90# having the poorest high-temperature stability. It is evident that under a stress of 3.2 kPa, the creep recovery rate of modified asphalt decreases nonlinearly due to the synergistic effect of solubility and temperature. This is attributed to the enhanced elastic dissipation effect caused by the disintegration of the three-dimensional network structure of the rubber powder. The activation process increases the free volume fraction of polymer segments, driving the viscoelastic behavior to shift from entropy-elastic to viscous flow.

(2) Irreversible Creep Modulus J_nr_

**Figure 8:**
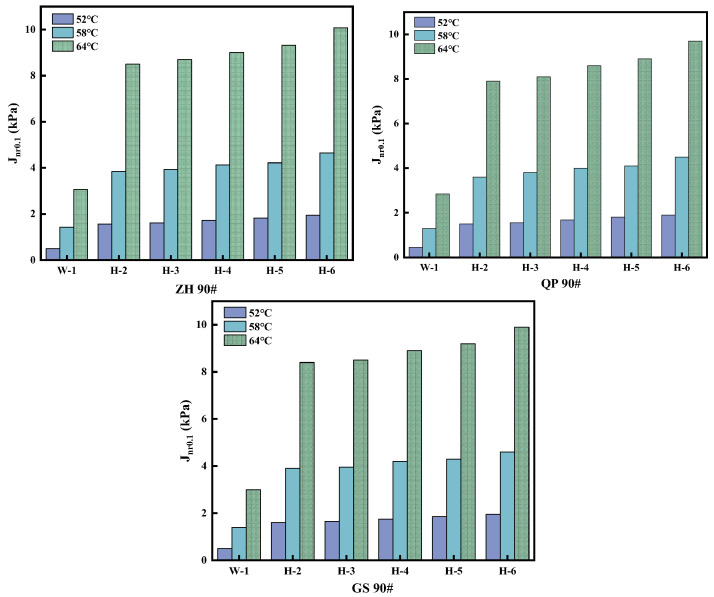
J_nr0.1_ value of activated rubber powder-modified asphalt.

The irreversible creep modulus J_nr_ is a key parameter for quantitatively evaluating the degree of plastic flow in asphalt; an increase in its value shows a significant negative correlation with the material’s resistance to deformation at high temperatures. Figure 8 shows the Jnr test results for various rubber powder-modified asphalts under different stress conditions.

The measured J_nr_ and R values were compared with the specification limits defined in AASHTO M332-18. According to this specification, the maximum allowable J_nr_ at 3.2 kPa for standard traffic (S) grade is 4.0 kPa^−1^; for heavy (H) grade, it is 2.0 kPa^−1^; for very heavy (V) grade, it is 1.0 kPa^−1^; and for extreme (E) grade, it is 0.5 kPa^−1^. At 64 °C, only the unactivated sample W-1 of QP 90# modified asphalt exhibited Jnr values approaching the S-grade limit, while most activated samples showed Jnr values exceeding 4.0 kPa^−1^. This indicates that while twin-screw extrusion activation improves rubber-asphalt compatibility, it significantly reduces high-temperature deformation resistance, necessitating further optimization of the activation process to achieve a balance between compatibility and rutting resistance. The corresponding R values were generally below the minimum recommended thresholds for all traffic grades at temperatures above 58 °C.

As shown in Figure 8, the Jnr_3.2_ values of all three modified asphalts increase significantly with rising temperature, and the activated samples generally exhibit higher values than the unactivated W-1 samples. Specifically, for ZH 90# modified asphalt at 52 °C, the J_nr3.2_ value of W-1 is 0.5 kPa^−1^, which increases to 1.5–2 kPa^−1^ after activation. When the temperature rose to 58 °C and 64 °C, the J_nr3.2_ values of W-1 increased to 1.5 kPa^−1^ and 3 kPa^−1^, respectively, and further increased to 3–4 kPa^−1^ and 8–10 kPa^−1^ after activation, indicating that the dissociation of the cross-linked network intensified at high temperatures, resulting in a significant reduction in deformation resistance. The trend of the J_nr3.2_ value for QP 90# at various temperatures is consistent with that of ZH 90#, but the values are slightly lower, indicating relatively better high-temperature rheological stability. The J_nr3.2_ value range for GS 90# is similar to that of QP 90#, but the viscosity response in the high-temperature range is more pronounced, and the rate of decline in deformation resistance is comparable to that of ZH 90#. Therefore, the activation process intensifies the dissociation of the rubber powders’ cross-linked network by increasing solubility, leading to an increase in the Jnr_3.2_ value and a weakening of high-temperature deformation resistance; QP 90#, due to its strong molecular chain restructuring ability, exhibits the slowest rate of high-temperature performance degradation and the best overall performance.

**Figure 9:**
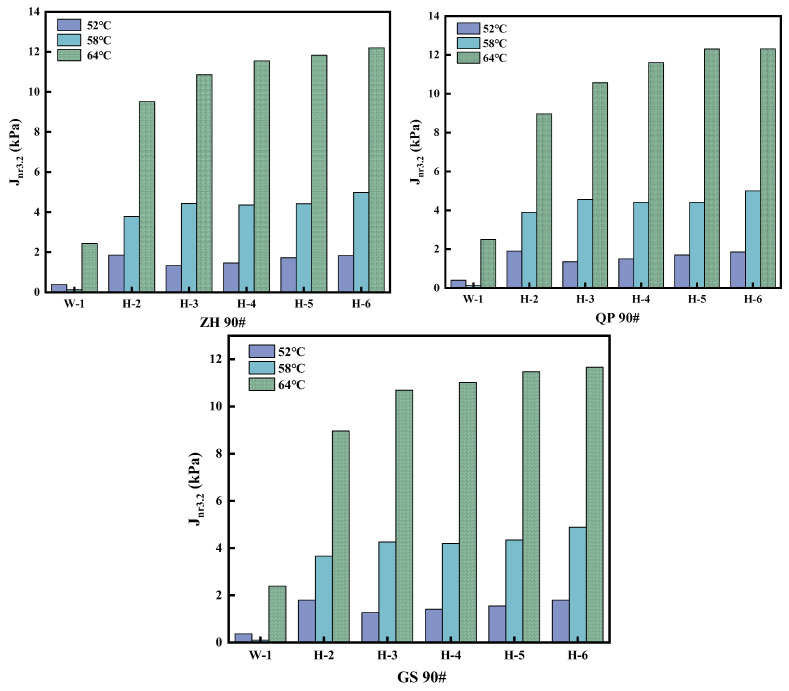
J_nr3.2_ value of activated rubber powder-modified asphalt.

As shown in Figure 9, the J_nr3.2_ values of all three modified asphalts exhibit distinct thermally induced viscoelastic decay characteristics, and the J_nr3.2_ values corresponding to the activated rubber powder are generally higher than those of the unactivated sample W-1. Specifically, the J_nr3.2_ value of ZH 90# modified asphalt after activation at 64 °C reaches 10–12 kPa^−1^, indicating the most severe deterioration in deformation resistance. The J_nr3.2_ of QP 90# modified asphalt after activation at the same temperature was also 10–12 kPa^−1^, similar to that of ZH 90#, but this value was slightly lower below 58 °C, and the decay rate was relatively gentle. For GS 90# modified asphalt, the J_nr3.2_ value after activation at 64 °C is 8–10 kPa^−1^, which, although lower than that of ZH 90#, still exhibits dominant viscous flow, resulting in weaker deformation resistance compared to QP 90#. In summary, the activation process generally weakens the material’s high-temperature deformation resistance. QP 90# exhibits the best high-temperature performance due to its excellent cross-linking stability and molecular chain synergy, while ZH 90# and GS 90# require optimization in terms of network stability and interfacial compatibility, respectively, to improve their rheological behavior.

As shown in Figure 8 and Figure 9, as temperature increases, the J_nr_ value of the asphalt continues to rise, high-temperature stiffness decreases, and the ability to resist external loads weakens. At different stress levels, increasing the solubility of the rubber powder causes the Jnr value of the modified asphalt to rise, indicating that the activated rubber powder increases the viscous components within the system, preventing complete recovery from deformation and consequently leading to deterioration in high-temperature performance. Based on a comprehensive evaluation of multiple factors, including creep recovery rate, irreversible creep modulus, and rubber powder activation degree, the QP 90# activated rubber powder-modified asphalt exhibits the best stability and resistance to deformation under high-temperature conditions. ZH 90# activated rubber powder modified asphalt has a lower high-temperature recovery rate and the weakest rutting resistance; although GS 90# has certain advantages, its high-temperature performance still falls short of QP 90#. Therefore, QP 90# activated rubber powder modified asphalt possesses superior high-temperature deformation resistance and rutting resistance, making it the optimal choice for road construction projects in high-temperature or rutting-prone areas.

### 3.3. Frequency Sweep Analysis

(1) Complex Modulus

**Figure 10:**
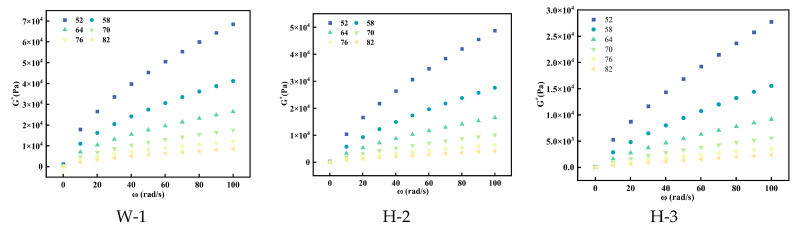

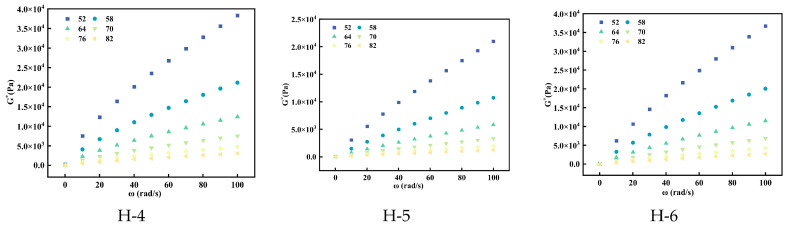
Complex modulus plot of ZH 90# modified asphalt.

**Figure 11:**
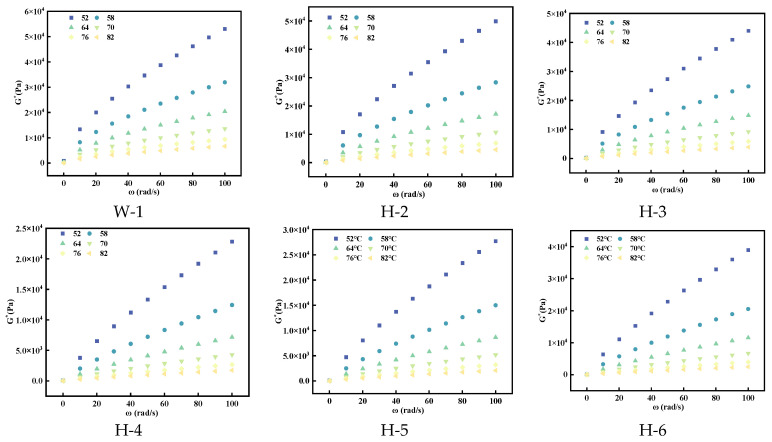
Complex modulus plot of QP 90# modified asphalt.

**Figure 12:**
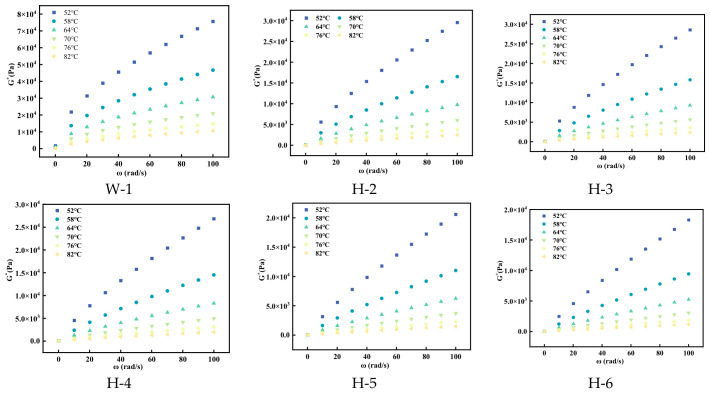
Complex modulus plot of GS 90# modified asphalt.

This analysis uses the complex modulus measured for ZH 90# modified asphalt during a frequency sweep from 0.1 to 100 rad/s as an example; the specific data are shown in Figure 10, Figure 11 and Figure 12.

As shown in Figure 10, the viscoelastic behavior of ZH 90# modified asphalt exhibits a distinct frequency dependence. Within the angular frequency scan range of 0.1–100 rad/s, the complex modulus increases significantly with rising frequency, rising from 10^4^ to 10^5^ Pa in the low-frequency range to 10^7^–10^8^ Pa in the high-frequency range. This change is consistent with the rheological behavior revealed by the time-temperature equivalence principle. The moduli of the various samples are relatively close in the low-frequency region, indicating that the material primarily exhibits Newtonian fluid characteristics. When the frequency increases to 10–100 rad/s, the maximum difference in modulus among formulations can reach one order of magnitude. Analysis of data dispersion also revealed that as the frequency passes through the viscoelastic transition region (1–10 rad/s), the standard deviation of the phase angle increases to ±1.5°. This phenomenon stems from the coupling between the sensitivity threshold of the testing system and the nonlinear response of the material’s internal structural relaxation processes.

A comparison of Figure 10, Figure 11 and Figure 12 shows that the complex modulus of all types of rubber powder-modified asphalt increases with rising angular frequency. The addition of rubber powder reduces the complex modulus of modified asphalt in the medium-temperature range, and as the solubility increases, the rate of decrease in modulus systematically increases, indicating that rubber powder has an adverse effect on deformation resistance under medium-temperature conditions. However, under higher temperature conditions, rubber powder actually enhances the complex modulus of modified asphalt. In particular, when the solubility reaches 40.02%, it not only fails to negatively affect high-temperature deformation resistance but actually slightly improves it.

(2) Phase angle (*δ*)

**Figure 13:**
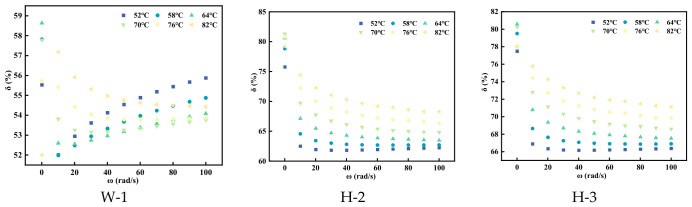

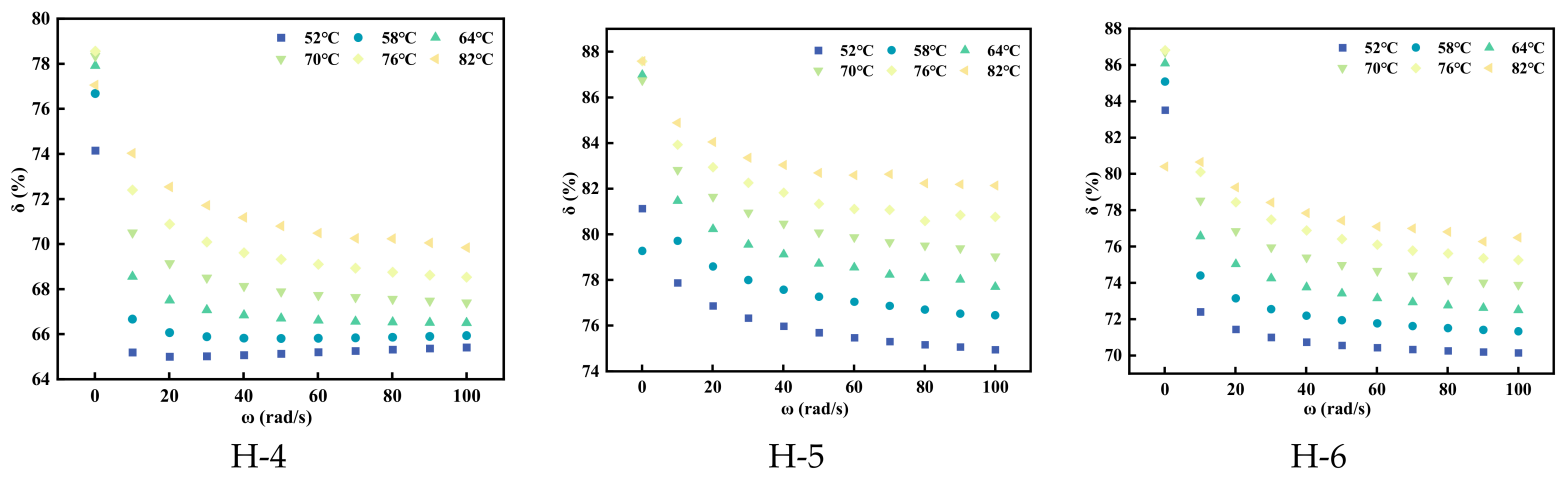
Phase angle of ZH 90# modified asphalt.

**Figure 14:**
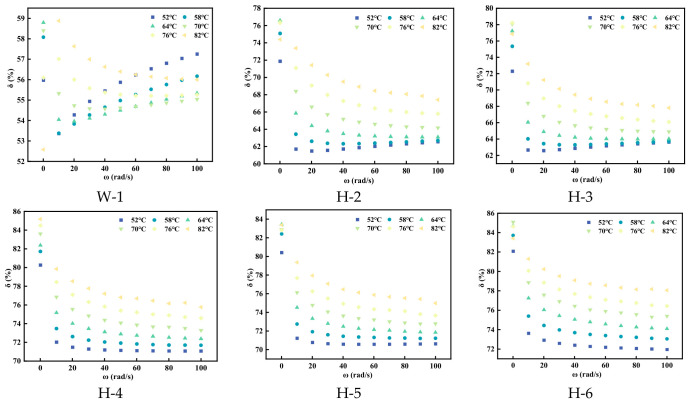
Phase angle of QP 90# modified asphalt.

**Figure 15:**
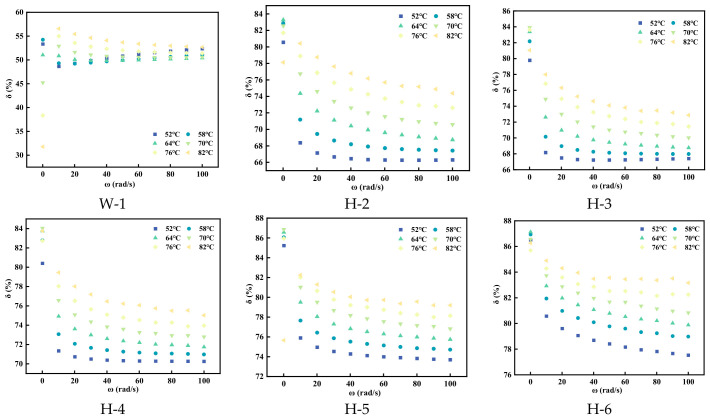
Phase angle of GS 90# modified asphalt.

Figure 13, Figure 14 and Figure 15 show that the phase angle of all rubber powder-modified asphalt samples decreases as the angular frequency increases. Specifically, the phase angle of ZH 90# modified asphalt generally increases with rising temperature, reflecting an increase in the proportion of viscous components in the material. However, as the frequency increases, the phase angle generally decreases, demonstrating certain elastic response characteristics. Among these, the H-5 and H-6 samples exhibit the largest phase angles at high temperatures, indicating strong temperature sensitivity and viscous-dominated behavior. In contrast, the phase angle of QP 90# modified asphalt remained at a moderate level, exhibiting good temperature stability and a more balanced viscoelastic behavior. GS 90# modified asphalt had a relatively lower phase angle under high-temperature conditions, demonstrating superior elastic recovery and resistance to high-temperature deformation.

Overall, the incorporation of rubber powder significantly reduced the phase angle of the modified asphalt, and this reduction increased further as the solubility of the rubber powder increased, particularly in the low-frequency range. This indicates that rubber powder can enhance the elasticity of modified asphalt and improve its elastic deformation capacity, with the effect being particularly pronounced under low-frequency conditions.

### 3.4. Black Diagrams

**Figure 16:**
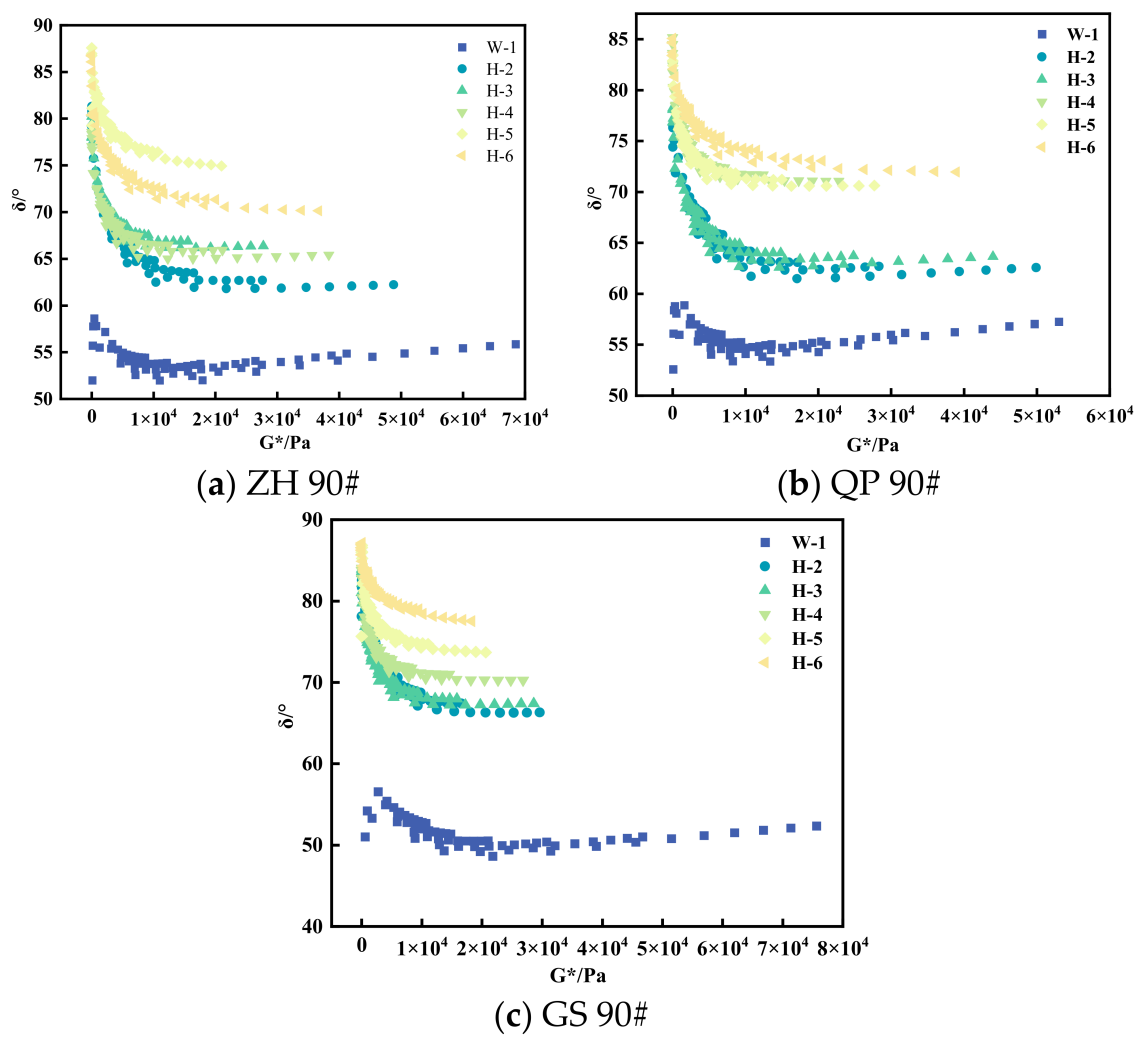
Black Curve.

To eliminate the temperature and frequency dependencies of the viscoelastic parameters, a graph is plotted with the phase angle on the vertical axis and the complex modulus on the horizontal axis; the resulting graph is known as a Black plot (also referred to as a box plot) [33]. Figure 16 shows the Black plots for various rubber powder-modified asphalts.

As shown in Figure 16, the Black curve for rubber powder-modified asphalt with poor compatibility and insufficient storage stability appears as a continuous curve. The Black curves for different modified asphalts vary in shape. In the coordinate plane defined by the complex modulus and phase angle, the curve reflects the viscoelastic properties of the material under different conditions. Generally, the slope of the curve and its trend are jointly influenced by the solubility of the rubber powder and the properties of the base asphalt. The Black curve of ZH 90# modified asphalt has a relatively small slope in the low complex modulus region, which gradually steepens as the modulus increases. This indicates that viscoelastic behavior changes little under low stress or strain levels, whereas the viscoelastic response is more pronounced under high stress or strain conditions. Furthermore, differences in rubber powder solubility result in varying phase angles corresponding to the same modulus. Modified asphalt with higher-solubility rubber powder typically exhibits a larger phase angle at the same modulus, indicating a higher proportion of viscous components.

### 3.5. Complex Modulus Master Curves

**Figure 17:**
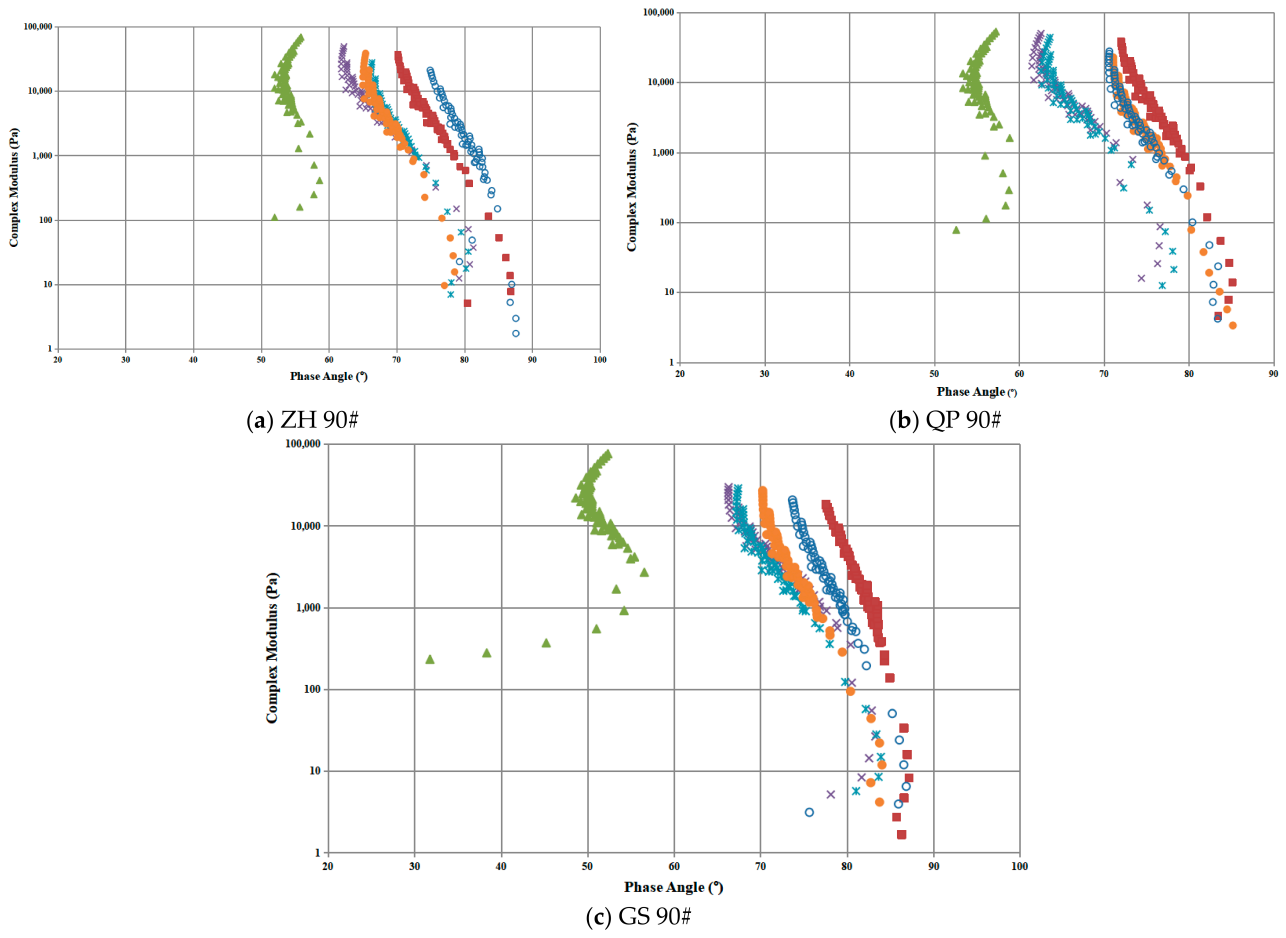
Main curve of the complex modulus of activated rubber powder-modified asphalt. Note: Green: W-1; Purple: H-2; Sky blue: H-3; Orange: H-4; Blue-purple: H-5; Red: H-6.

The complex modulus master curves were constructed using the time-temperature superposition principle (TTSP) at a reference temperature of 25 °C. Frequency sweep data obtained at multiple temperatures (ranging from 40 °C to 76 °C) were horizontally shifted along the logarithmic frequency axis to form a continuous master curve spanning 10^−2^ to 10^8^ Hz. The shifting procedure was performed using TA Universal Analysis software (version 5.2, TA Instruments, New Castle, DE, USA), and the quality of the master curve construction was verified by the smoothness of the resulting curves and the absence of discontinuities. The master curves are shown in Figure 17.

The complex modulus master curves were constructed using the time-temperature superposition principle (TTSP) at a reference temperature of 25 °C. Frequency sweep data obtained at multiple temperatures (ranging from 40 °C to 76 °C) were horizontally shifted along the logarithmic frequency axis to form a continuous master curve spanning 10^−2^ to 10^8^ Hz. The shift factors αT were determined using the Williams-Landel-Ferry (WLF) Equation (15):

(15) $$\log_{\alpha T}=-\frac{C_{1}\left(T-T_{0}\right)}{C_{2}+\left(T-T_{0}\right)}$$

where *T*_0_ is the reference temperature (25 °C), and *C*_1_ and *C*_2_ are material-specific constants determined by nonlinear least-squares regression. The shifting procedure was performed using TA Instruments TRIOS software (version 5.1.1, TA Instruments, New Castle, DE, USA), and the quality of the master curve construction was verified by the smoothness of the resulting curves and the absence of discontinuities. The master curves are shown in Figure 17.

Figure 17 shows the main curves of modified bitumen using different activated rubber powders at 25 °C.

At a reference temperature of 25 °C, the complex modulus curves of asphalt modified with different activated rubber powders exhibit the following patterns: in the low-frequency range, the modulus decreases rapidly as frequency decreases, while in the high-frequency range, the modulus increases gradually as frequency increases. As shown in Figure 17a, ZH 90# modified asphalt exhibits typical viscous fluid behavior in the low-frequency range, with a phase angle close to 90° and an exponential decay of the complex modulus as frequency decreases, indicating that the molecular segments have achieved sufficient relaxation. When the frequency exceeds the critical value of 10 rad/s for the viscoelastic transition, the system shifts to being dominated by the storage modulus, with G* rising to the order of 10^7^ Pa, exhibiting glassy elastic characteristics. Figure 17b shows that although QP 90# modified asphalt maintains a similar rheological trend, its modulus decay rate is slightly lower than that of ZH 90#, indicating that its binder network possesses a higher entanglement density. In contrast, Figure 17c shows that the modulus of GS 90# modified bitumen decreases most rapidly in the low-frequency region, with the phase angle remaining above 85°, reflecting restricted molecular chain motion due to insufficient swelling of the rubber powder. At the same time, the increase in its storage modulus in the high-frequency range (>50 rad/s) tends to slow down, which is related to the gradient structure of the interfacial transition layer. The critical frequency shift observed in all three cases quantitatively characterizes the regulatory effect of differences in rubber powder solubility on the kinetics of the viscoelastic transition.

A comparative analysis of dynamic shear rheological properties revealed significant differences in the viscoelastic characteristic curves of various modified asphalts. Under identical frequency conditions, the complex modulus G* of ZH 90# modified bitumen was significantly higher than that of QP 90# and GS 90#, particularly in the high-frequency range, where the difference in modulus could reach 0.5 to 1 order of magnitude, indicating that ZH 90# possesses superior elastic recovery capability under high-shear loads. This result is closely related to the solubility parameters in the rubber powder modification system; the lower solubility of rubber powder in ZH 90# modified asphalt leads to a denser three-dimensional network structure, thereby enhancing the material’s resistance to shear deformation. Further analysis of the main curve morphology reveals that ZH 90# modified asphalt exhibits significant nonlinear growth in the 0.1–10 rad/s frequency range, with the critical frequency of its viscoelastic transition zone shifting to the left by approximately 0.8 logarithmic cycles relative to the reference sample, reflecting the phenomenon of an earlier glass transition caused by the hindrance of activated rubber powder molecular segment motion. In contrast, the modulus of QP 90# and GS 90# modified bitumen varies more gradually with frequency, indicating a more thorough compatibility reconstruction between the rubber powder phase and the bitumen matrix, resulting in the formation of a gradient-type interfacial transition layer. The fundamental cause of the aforementioned differences in rheological response lies in the regulatory effect of rubber powder activation process parameters (such as shear stress and activation time) on the multiscale structure of modified asphalt (degree of swelling, crosslinking density, and entanglement network). These characteristics can be quantitatively evaluated using the principal curve of the energy storage modulus constructed based on the superposition principle of time and temperature.

It should be noted that while ZH 90# modified asphalt exhibited higher complex modulus values under certain frequency and temperature conditions, the comprehensive evaluation of multiple rheological parameters, including the decay pattern of G*, phase angle evolution, creep recovery rate, and irreversible creep compliance, consistently identified QP 90# modified asphalt as possessing the most balanced and stable high-temperature performance. The superiority of QP 90# lies in its optimal viscoelastic balance and structural stability, rather than in any single parameter.

## 4. Conclusions

In this study, desulfurized rubber powder with different solubilities was prepared using a twin-screw extrusion process. Using three types of base bitumen, QP 90#, ZH 90#, and GS 90#, as the matrix, the effects of rubber powder solubility on the high-temperature rheological and viscoelastic properties of modified bitumen were systematically investigated using methods such as DSR, MSCR, and frequency scanning. Through a comparative analysis of the high-temperature rheological properties of the three base asphalts and the modified asphalts containing rubber powders of different solubilities, the following conclusions were drawn:

(1) Twin-screw extrusion desulfurization effectively breaks S-S crosslinks in crumb rubber, increases CR surface activity, and improves CR-asphalt compatibility. Desulfurization allows controlled adjustment of crosslink density, significantly enhancing high-temperature rheological properties. However, over-desulfurization leads to main chain scission and weakens high-temperature deformation resistance.

(2) Among the three modified asphalts, QP 90# exhibits the mildest attenuation of G* with temperature, the smallest increase in phase angle, and the best high-temperature stability of the rutting factor. ZH 90# and GS 90# show more pronounced performance deterioration over different temperature ranges, with ZH 90# having particularly strong viscous flow at high temperatures.

(3) MSCR results show that QP 90# has the highest creep recovery rate R and the lowest non-recoverable creep compliance Jnr at both 0.1 kPa and 3.2 kPa. At 64 °C, the R_3.2_ of QP 90# remains positive, while those of ZH 90# and GS 90# drop to zero or become negative, indicating that QP 90# has significantly better permanent deformation resistance and elastic recovery at high temperatures.

(4) Black diagrams and complex modulus master curves confirm that QP 90# has the best CR-asphalt compatibility, with a smooth and regular Black diagram and the gentlest frequency dependence of the master curve. A gradient interfacial transition layer is formed. This binder has excellent viscoelastic balance and structural stability, making it suitable for high-temperature and rainy regions.

(5) CR solubility critically influences high-temperature rheological behavior. Moderate desulfurization balances elastic and viscous components and optimizes viscoelastic response. Excessive desulfurization causes main chain scission, loss of mechanical strength, and deterioration of high-temperature deformation resistance. In engineering applications, solubility should be selected based on the service environment, crosslink density, and rheological response; a moderate solubility is recommended.

It should be noted that the current study focuses on the high-temperature rheological properties of desulfurized rubber powder-modified asphalt under unaged conditions. In real pavement applications, factors such as oxidative aging, fatigue damage, moisture intrusion, and environmental exposure will inevitably affect the long-term performance of the material. Future studies will incorporate accelerated aging tests using the pressure aging vessel (PAV), fatigue characterization via the linear amplitude sweep (LAS) test, and moisture susceptibility evaluation to provide a more comprehensive understanding of the field performance of desulfurized rubber powder-modified asphalt. Additionally, field validation studies, including test sections and long-term performance monitoring, will be conducted to establish correlations between laboratory rheological parameters and actual pavement performance.

## Figures and Tables

**Table 1:** Waste rubber powder basic technical indexes.

Parameter (%)	Specification	Measured Value
Iron content	≤0.05	0.04
Residue (40 mesh)	≤10	7.0
Water content	≤1.0	0.54
Heating loss	≤1.0	0.8
Rubber hydrocarbon	≥45	52
Ash	≤10	8.1
Carbon black	≥26	35
Acetone extract	≤8	6

Note: All technical indicators meet the requirements of Ground vulcanized rubber (GB/T 19208-2020) [19].

**Table 2:** Technical indicators of QP 90# asphalt.

Parameter		Measured	Specification
Softening point (°C)		46.5	≥42
Penetration (0.1 mm)		84.7	80~100
Ductility (cm)		>100	≥100
RTFOT (163 °C, 85 min)	Penetration ratio (%)	61	≥54
	Ductility at 10 °C (cm)	9.3	≥6.0
	Mass loss (%)	0.11	≤±0.8

## Data Availability

The original contributions presented in this study are included in the article. Further inquiries can be directed to the corresponding authors.
